# Initial Outcomes of Transdiagnostic Internet-Delivered Cognitive Behavioral Therapy Tailored to Public Safety Personnel: Longitudinal Observational Study

**DOI:** 10.2196/27610

**Published:** 2021-05-05

**Authors:** Heather D Hadjistavropoulos, Hugh C McCall, David L Thiessen, Ziyin Huang, R Nicholas Carleton, Blake F Dear, Nickolai Titov

**Affiliations:** 1 Department of Psychology University of Regina Regina, SK Canada; 2 Department of Mathematics and Statistics University of Regina Regina, SK Canada; 3 PSPNET University of Regina Regina, SK Canada; 4 eCentreClinic Department of Psychology Macquarie University Sydney Australia

**Keywords:** internet, cognitive behavior therapy, anxiety, depression, posttraumatic stress disorder, public safety personnel, CBT, internet-based cognitive behavioral therapy, ICBT, PTSD, outcome, diagnosis, longitudinal, observational, literature, effectiveness

## Abstract

**Background:**

Canadian public safety personnel (PSP) experience high rates of mental health disorders and face many barriers to treatment. Internet-delivered cognitive behavioral therapy (ICBT) overcomes many such barriers, and is effective for treating depression, anxiety, and posttraumatic stress disorder (PTSD) symptoms.

**Objective:**

This study was designed to fill a gap in the literature regarding the use of ICBT tailored specifically for PSP. We examined the effectiveness of a tailored ICBT program for treating depression, anxiety, and PTSD symptoms among PSP in the province of Saskatchewan.

**Methods:**

We employed a longitudinal single-group open-trial design (N=83) with outcome measures administered at screening and at 8 weeks posttreatment. Data were collected between December 5, 2019 and September 11, 2020. Primary outcomes included changes in depression, anxiety, and PTSD symptoms. Secondary outcomes included changes in functional impairment; symptoms of panic, social anxiety, and anger; as well as treatment satisfaction, working alliance, and program usage patterns.

**Results:**

Clients reported large symptom reductions on measures of depression and anxiety, as well as moderate reductions on measures of PTSD and secondary symptoms, except for social anxiety. Most clients who reported symptoms above clinical cut-offs on measures of depression, anxiety, and PTSD during screening experienced clinically significant symptom reductions. Results suggested good engagement, treatment satisfaction, and working alliance.

**Conclusions:**

Tailored, transdiagnostic ICBT demonstrated promising outcomes as a treatment for depression, anxiety, and PTSD among Saskatchewan PSP and warrants further investigation.

**Trial Registration:**

Clinicaltrials.gov NCT04127032; https://www.clinicaltrials.gov/ct2/show/NCT04127032

## Introduction

Public safety personnel (PSP) include border security personnel, communications officials (eg, emergency response dispatchers), correctional workers, firefighters, paramedics, police officers, and others whose work keeps communities safe [1]. Through their work, PSP frequently experience or witness potentially traumatic events such as motor vehicle collisions and acts of violence [2-4]. Research shows that such events put individuals at risk of developing several emotional disorders, including posttraumatic stress disorder (PTSD), major depressive disorder, and panic disorder [5-10]. In Canada, a large survey study indicated that 44.5% of PSP report clinically significant symptoms of one or more mental health disorders [11]. PSP have also reported high rates of mental health problems in other countries [12-14]. PSP face many barriers to treatment, such as stigma, distance from services, and time constraints [15]. Stigma appears to be a particularly prohibitive barrier to treatment among PSP [15,16].

Internet-delivered cognitive behavioral therapy (ICBT) is a treatment wherein traditional cognitive behavioral therapy skills are taught via online learning platforms and is poised to help overcome these barriers to treatment for PSP. ICBT can be accessed privately and conveniently, and it is cost-effective because it requires less therapist time per client than traditional face-to-face treatments [17,18]. Meta-analyses have shown that ICBT is effective for treating symptoms of a range of mental disorders, including depression, anxiety, and PTSD [19,20]. There have been several recent calls for internet interventions to be tailored to the unique needs of their target users to facilitate greater treatment engagement [21,22]. As one group stated, this process begins with establishing “a deep understanding of user needs and preferences, and actively involving users in design processes from the outset” [21]. Tailoring ICBT may be particularly important for PSP, who have reported several unique needs such as the need for flexible treatment timelines, diverse preferences for frequency of therapist support, specialized therapists with knowledge of unique PSP issues and culture, and a focus on exposures to potentially psychologically traumatic events [15]. Canadian PSP appear receptive to therapist-supported ICBT, which was ranked as their most preferred mental health treatment after face-to-face psychological services [23].

The Government of Canada recently announced a $10 million investment into developing, delivering, and evaluating ICBT tailored specifically for PSP [24]. Our research unit, PSPNET, was contracted to carry out this work. We conducted extensive interviews with PSP stakeholders to better understand their needs and preferences [15] and, drawing upon their feedback, tailored a transdiagnostic ICBT program that previously demonstrated positive outcomes for a range of conditions to meet the needs of PSP. The tailored course of treatment is called the PSP Wellbeing Course, which is described in detail below.

This paper presents the initial outcomes of the PSP Wellbeing Course among the first 83 PSP enrolled in the course during the first 7 months of availability in Saskatchewan. The study objectives were to evaluate the initial (a) effectiveness, (b) treatment satisfaction and working alliance, and (c) program usage patterns of the PSP Wellbeing Course. We hypothesized high completion rates, high satisfaction, and moderate to large effect sizes for symptom change. This study is the first, as far as we are aware, to evaluate the tailored ICBT approach for treating mental health problems among PSP.

This study’s sample represents only the first third of the sample described in our trial protocol (N=250; Clinicaltrials.gov NCT04127032). This early evaluation of outcomes is important for three reasons. First, it is important to ensure that the development and delivery of the PSP Wellbeing Course is a fruitful use of Canadian tax dollars. Second, evaluation of early outcomes is consistent with the learning health system approach, which is characterized by a continuous cycle of gathering data from practice, transforming data into knowledge, and implementing knowledge into practice [25]. Third, our preliminary findings may be helpful for others involved in the development, provision, evaluation, and funding of mental health services for PSP.

## Methods

### Study Design

We employed a longitudinal single-group open-trial design. This study was approved by the local institutional research ethics board (2019-157) and registered on Clinicaltrials.gov (NCT04127032). This article presents the initial study outcomes for the first 83 clients who enrolled in the PSP Wellbeing Course (one third of the total expected sample of 250). We have followed the STROBE (Strengthening the Reporting of Observational Studies in Epidemiology) statement in preparing the article [26].

### Setting

This study was performed in the Canadian province of Saskatchewan. There was no specialized ICBT for PSP in Saskatchewan prior to this study. ICBT has been available to Saskatchewan residents through the Online Therapy Unit [27] since 2010, but the numbers of PSP using this service have not been systematically tracked.

### Clients

All clients who enrolled in the PSP Wellbeing Course between December 5, 2019 and July 2, 2020 consented to our use of their data for research and were included in this study. Enrollment in the PSP Wellbeing Course required clients to (a) be 18 years or older, (b) currently or formerly employed as a PSP (as defined above), (c) have access to a computer and internet service, (d) provide an emergency medical contact, and (e) reside in the province of Saskatchewan (although we later began offering the PSP Wellbeing Course in English and French to residents of Quebec as well). Prospective clients were excluded and referred to appropriate services if they (a) reported a high suicide risk; (b) had attempted suicide or had been hospitalized for a high suicide risk within the past year; (c) reported a primary problem with psychosis, mania, or alcohol and drugs; (d) reported receiving psychological treatment more than twice per month; or (e) reported concerns about ICBT and requested referral to other services in the community.

### Intervention

The PSP Wellbeing Course was adapted from a transdiagnostic ICBT program called the Wellbeing Course, which was developed for use in the general population by eCentre Clinic at Macquarie University, Australia [28-32]. The PSP Wellbeing Course is designed to treat symptoms of depression, anxiety, and PTSD. The course consists of five sequential lessons, which are presented in a slideshow format and focus on (1) introducing the cognitive behavioral model and symptom identification, (2) monitoring and challenging maladaptive thoughts, (3) managing physical symptoms, (4) graded exposure, and (5) relapse prevention. Lesson content is relayed through instructive text, diagrams, and illustrative case stories about PSP. Clients are also given access to downloadable materials, weekly homework assignments, and supplementary resources related to issues not addressed in the lessons (eg, assertiveness, communication, sleep problems). Therapists hold graduate degrees in psychology or social work and provide support to clients by phone or secure email up to twice per week, depending on each client’s preference. Each week, clients complete symptom questionnaires and a reflection questionnaire including both open-ended and closed-ended questions designed to gauge response to the treatment program (eg, what clients worked on; any challenges they encountered; and ratings of helpfulness, understanding, and difficulty). The course is designed to be completed in 8 weeks, but clients are provided with therapist support for up to 16 weeks based on symptoms and preference, and are given access to the course materials for 1 year. We tailored the PSP Wellbeing Course using extensive feedback from PSP stakeholders gleaned through 112 interviews [15] and 132 survey responses [23] (eg, replacing generic case stories and examples used to illustrate treatment concepts with case stories and examples about PSP).

### Procedure

Prospective clients completed a two-stage eligibility screening process consisting of an online screening questionnaire (demographics, clinical history, and the measures described below) and a screening interview by phone. Clients then began the PSP Wellbeing Course. Clients were automatically administered brief weekly questionnaires and a longer battery of questionnaires at 8 weeks, as detailed below. Questionnaires were embedded in the course. Members of our research team followed up with clients by phone or email to encourage them to complete posttreatment measures.

### Measures

#### Primary Outcome Measures

##### Patient Health Questionnaire-9

The Patient Health Questionnaire-9 (PHQ-9) measures symptoms of depression and includes one item to assess suicidality [33]. It includes nine items rated from 0 (not at all) to 3 (nearly every day), with total scores ranging from 0 to 27. A score greater than 9 is typically used to identify a likely diagnosis of major depressive disorder [34]. The PHQ-9 has demonstrated excellent psychometric properties [33,35,36]. Cronbach α for the PHQ-9 in this study ranged from .84 to .88.

##### Generalized Anxiety Disorder-7

Generalized Anxiety Disorder-7 (GAD-7) is a brief measure of general anxiety symptoms [37]. It includes seven items rated from 0 (not at all) to 3 (nearly every day) for a total score ranging from 0 to 21. The GAD-7 has demonstrated strong psychometric properties, and a score of 10 or greater is typically used to identify a likely diagnosis of generalized anxiety disorder [36,37]. Cronbach α for the GAD-7 in this study ranged from .86 to .92.

##### Posttraumatic Stress Disorder Checklist for DSM-5

The Posttraumatic Stress Disorder Checklist for the DSM-5 (PCL-5) measures symptoms of PTSD and includes 20 items rated from 0 (not at all) to 4 (extremely) [38]. A cut-off of 33 was recommended by several groups to indicate a likely diagnosis of PTSD [39,40]. The PCL-5 has shown good psychometric properties [39]. Cronbach α for the PCL-5 in this study ranged from .95 to .96.

#### Secondary Outcome Measures

##### Panic Disorder Severity Scale Self-Report

The Panic Disorder Severity Scale Self-Report (PDSS-SR) is a psychometrically sound 7-item measure assessing symptoms of panic disorder [41]. Items are rated from 0 to 4 for a total score of 0 to 28. It is common practice to employ a cut-off score of 8 on the PDSS-SR [42-44], based on the cut-off initially established for the nonself-report PDSS [45]. Cronbach α for the PDSS-SR in this study was .89-.91.

##### Six-Item Social Phobia Scale and Six-Item Social Interaction Anxiety Scale

The 6-item Social Phobia Scale (SPS-6) and 6-item Social Interaction Anxiety Scale (SIAS-6) are brief measures of social anxiety that are psychometrically sound when administered together or separately [46]. Items are rated from 0 to 4 for total scores of 0 to 24 on each measure or a combined total of 0 to 48, with a clinical cut-off of 2 on the SPS-6 and of 7 on the SIAS-6 [46]. Cronbach α for the measures combined in the current study was .91 and .94.

##### Dimensions of Anger Reactions Scale

The Dimensions of Anger Reactions Scale (DAR-5) is a 5-item measure of anger that has demonstrated good psychometric properties [47]. Scores range from 5 to 25 with a cut-off of 12 [48]. Cronbach α for the DAR-5 in this study was .88 and .89.

##### Sheehan Disability Scale

The Sheehan Disability Scale (SDS) is a psychometrically sound 3-item measure assessing respondents’ functioning in their home lives, social lives, and work/school lives [49].

##### Treatment Satisfaction Questionnaire

We administered a bespoke questionnaire to explore treatment satisfaction.

##### Working Alliance Inventory-Short Revised

The Working Alliance Inventory-Short Revised (WAI-SR) is a 12-item measure of working alliance with goal, task, and bond subscales that has demonstrated good psychometric properties [50,51]. The Cronbach α for the WAI-SR in this study was .94 for the goal subscale, .93 for the task subscale, and .95 for the bond subscale.

#### Administration of Measures

At screening and posttreatment, clients were administered the PHQ-9, GAD-7, PLC-5, PDSS-SR, SPS-6, SIAS-6, DAR-5, and SDS. Additionally, at posttreatment, clients were administered the Treatment Satisfaction Questionnaire and WAI-SR. Certain measures were administered weekly during treatment, and at 6 months and 1 year postenrollment. These time points are not included in the present analyses but will be reported in the future once more data are available.

### Analyses

Following intention-to-treat principles, we first created multiple imputations for missing data using predictive mean matching. Imputations for missing data were modeled controlling for lesson completion rates, pretreatment symptom measures, and, if available, weekly symptom measures. We used generalized estimating equations (GEEs) to model the change in symptoms from pretreatment to posttreatment [52]. A gamma distribution with a log-link function was used to model changes between pretreatment and posttreatment as proportional and to accommodate the skewed response distribution [53]. We ran the imputations and GEE models in R version 4.0.3 using the mice [54] and geepack [55] packages.

The effectiveness of the PSP Wellbeing Course was evaluated by examining Hedges *g* and proportional reductions in symptoms. Hedges *g* is calculated as the difference in means between pretreatment and posttreatment divided by the pooled SD and multiplied by a small-sample bias correction. It is interpreted similarly to Cohen *d* but was considered to be a more appropriate measure of effect size for this study given the small size of our clinical subsamples. Some researchers have recommended calculating Hedges *g* using the SD of the control or pretreatment group instead of the pooled SD when pretreatment and posttreatment SDs are unequal [56]. Indeed, we noted that in the subsets of clients scoring above clinical cut-offs at pretreatment, the pretreatment SDs were much lower than the posttreatment SDs. However, because these subsets represented a restricted range of pretreatment scores, a greater SD at posttreatment would be expected—even in the absence of a treatment effect—due to regression toward the mean [57]. Therefore, we used pooled SDs in calculating Hedges *g* in the clinical subsets to avoid overestimating effect sizes.

We also examined clinically significant deterioration and recovery for the PHQ-9, GAD-7, and PCL-5. Following the UK National Health Service guidelines, deterioration was defined as an increase of at least 6 points on the PHQ-9 [58]. Following precedents set in previous research using the same outcome measures, deterioration was defined as an increase of 5 points on the GAD-7 [59,60] or 10 points on the PCL-5 [61]. Recovery was defined as a decrease of at least 6 points on the PHQ-9, 5 points on the GAD-7, or 10 points on the PCL-5 that resulted in the patient moving from the clinical range to the nonclinical cut-off scores described in the Measures section above. We examined the uptake, treatment satisfaction and acceptability, and program usage patterns using descriptive statistics in SPSS version 23.

## Results

### Client Characteristics

**Figure 1:**
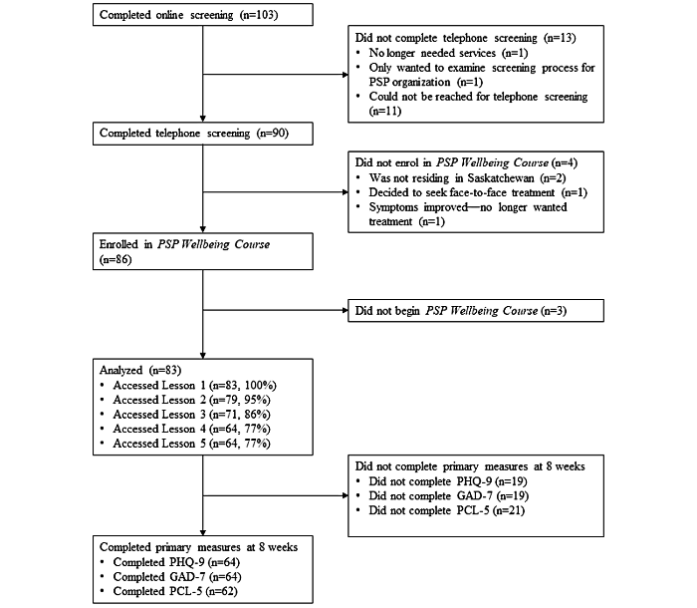
Flowchart displaying client enrollment, program usage, and completion of posttreatment measures. GAD-7: Generalized Anxiety Disorder-7; PCL-5: Posttraumatic Stress Disorder Checklist for the DSM-5; PHQ-9: Patient Health Questionnaire-9; PSP: public safety personnel.

There were 83 clients included in this study. The flow of clients through the study process is shown in Figure 1. Client characteristics are summarized in Table 1.

**Table 1:** Client demographics (N=83).

Characteristic			Value
Age (years), mean (SD)			40.25 (10.95)
**Gender, n (%)**			
	Woman	45 (54)	
	Man	37 (45)	
	Nonbinary	1 (1)	
**Ethnicity, n (%)**			
	White	73 (88)	
	First Nations, Inuit, or Metis	7 (8)	
	Other	3 (4)	
**Married or common law, n (%)**			
	Yes	53 (64)	
	No	30 (36)	
**Children, n (%)**			
	Yes	44 (53)	
	No	39 (47)	
**Community size, n (%)**			
	Population of 100,000 or greater	43 (52)	
	Population under 100,000	40 (48)	
**PSP^a^ sector, n (%)**			
	Police	24 (29)	
	Corrections	9 (11)	
	Emergency medical service	29 (35)	
	Fire	6 (7)	
	Dispatch/communications	5 (6)	
	Other	10 (12)	

^a^PSP: public safety personnel.

### Effectiveness

The effect sizes for each scale, along with the percentage changes and reliable change rates for primary symptom measures are detailed in Table 2. The results indicated large effect sizes on the PHQ-9 and GAD-7, and a moderate effect size on the PCL-5. When examining effect sizes for each primary measure only among clients reporting clinically significant symptoms on that measure at screening, we found large effect sizes on all three measures. In general, we found moderate effect sizes on secondary symptom measures in the entire sample and moderate to large effect sizes when examining only clients reporting clinically significant symptoms at screening; however, the results indicated small effect sizes on the combined SIAS-6 and SPS-6 among all clients and for those with clinically significant SIAS-6 and SPS-6 scores at screening.

**Table 2:** Effectiveness of the PSP Wellbeing Course.

Questionnaire		Pretreatment, mean (SD)	Posttreatment, mean (SD)	Hedges *g* effect size (95% CI) from pretreatment	Percentage change (95% CI) in estimated mean from pretreatment	Reliable deterioration (%)	Reliable recovery (%)
**Total sample (N=83)**							
	PHQ-9^a^	11.09 (5.59)	6.81 (5.33)	0.78 (0.46 to 1.09)	39 (26 to 49)	4.4	29.7
	GAD-7^b^	10.32 (5.44)	6.01 (4.82)	0.83 (0.52 to 1.15)	42 (30 to 52)	1.7	37.6
	PCL-5^c^	29.82 (19.18)	20.39 (17.60)	0.51 (0.20 to 0.82)	32 (15 to 45)	6.8	25.1
	PDSS-SR^d^	5.20 (5.09)	3.17 (4.52)	0.42 (0.11 to 0.73)	39 (15 to 56)	N/A^e^	N/A
	SIAS-6/SPS-6^f^	9.76 (9.22)	9.19 (9.58)	0.06 (–0.24 to 0.36)	6 (–20 to 26)	N/A	N/A
	DAR-5^g^	10.31 (4.39)	8.59 (3.79)	0.42 (0.11 to 0.72)	17 (8 to 25)	N/A	N/A
	SDS^h^ Family	4.98 (2.73)	3.69 (2.84)	0.46 (0.15 to 0.77)	26 (10 to 39)	N/A	N/A
	SDS Social	5.03 (2.94)	3.82 (2.92)	0.41 (0.10 to 0.72)	24 (9 to 37)	N/A	N/A
	SDS Work	4.54 (3.02)	3.40 (3.05)	0.37 (0.07 to 0.68)	25 (7 to 40)	N/A	N/A
	SDS Total	14.54 (7.81)	10.80 (7.76)	0.48 (0.17 to 0.79)	26 (11 to 38)	N/A	N/A
**Clinical sample^i^**							
	PHQ-9 (≥10, n=48)	14.90 (3.82)	8.91 (5.46)	1.25 (0.82 to 1.70)	40 (28 to 51)	2.9	51.3
	GAD-7 (≥10, n=46)	14.22 (3.24)	8.08 (5.11)	1.41 (0.97 to 1.87)	43 (31 to 53)	2.5	56.8
	PCL-5 (≥33, n=35)	48.63 (10.66)	32.96 (17.63)	1.05 (0.56 to 1.56)	32 (17 to 45)	7.8	59.5
	PDSS-SR (≥8, n=23)	12.14 (2.94)	7.00 (6.18)	1.03 (0.42 to 1.66)	42 (15 to 61)	N/A	N/A
	SIAS-6/SPS-6 (SIAS≥7 SPS≥2, n=27)	20.52 (7.54)	18.48 (10.23)	0.22 (–0.31 to 0.76)	10 (–13 to 28)	N/A	N/A
	DAR-5 (≥12, n=25)	15.84 (3.21)	11.76 (4.80)	0.97 (0.39 to 1.57)	26 (12 to 37)	N/A	N/A
	SDS Family (≥3, n=66)	6.03 (1.93)	4.23 (2.84)	0.73 (0.38 to 1.09)	30 (15 to 42)	N/A	N/A
	SDS Social (≥3, n=62)	6.34 (2.11)	4.45 (2.88)	0.74 (0.38 to 1.11)	30 (16 to 42)	N/A	N/A
	SDS Work (≥3, n=58)	5.98 (2.40)	4.23 (3.00)	0.64 (0.27 to 1.01)	29 (13 to 43)	N/A	N/A
	SDS Total (≥9, n=62)	17.95 (5.74)	12.35 (7.75)	0.81 (0.45 to 1.18)	31 (17 to 43)	N/A	N/A

^a^PHQ-9: Patient Health Questionnaire-9.

^b^GAD-7: Generalized Anxiety Disorder Questionnaire-7.

^c^PCL-5: Posttraumatic Stress Disorder Checklist for DSM-5.

^d^PDSS-SR: Panic Disorder Severity Scale Self-Report.

^e^N/A: not applicable; reliable recovery and deterioration were only calculated for primary measures because the magnitude of the score change required to determine reliable score changes for several secondary measures was unknown.

^f^SIAS-6/SPS-6: 6-item Social Interaction Anxiety Scale/6-item Social Phobia Scale.

^g^DAR-5: Dimensions of Anger Reactions-5.

^h^SDS: Sheehan Disability Scale.

^i^For each measure, estimated means, effects sizes, and percentage change are calculated based on those who scored in the clinical range on the measure at pretreatment.

### Treatment Satisfaction and Working Alliance

Of the 62 clients who completed the WAI-SR and questions related to treatment satisfaction, 54/62 (86%) indicated that the PSP Wellbeing Course had increased or greatly increased their confidence in their ability to manage their symptoms, and 61/62 (98%) indicated that the PSP Wellbeing Course was worth their time. Mean scores on the bond, goal, and task subscales of the WAI-SR were 16.11 (SD 4.64), 14.50 (SD 4.72), and 14.63 (SD 4.07), respectively. To our knowledge, there are no formal guidelines for interpreting WAI-SR scores, but these participant scores fall in the “high/positive” range according to the interpretation of one group of ICBT researchers [62].

### Program Usage Patterns

All 83 clients included in the analyses accessed Lesson 1 of the PSP Wellbeing Course. Most clients also accessed Lesson 2 (79/83, 95%), Lesson 3 (71/83, 86%), Lesson 4 (64/83, 77%), and Lesson 5 (64/83, 77%). Of the 60 clients who completed 8-week measures, at the time they did so, all had accessed Lesson 1 and Lesson 2, and most had accessed Lesson 3 (58/60, 97%), Lesson 4 (54/60, 90%), and Lesson 5 (45/60, 75%). Across the entire sample, most clients who accessed each lesson also accessed the accompanying DIY guides (92% or more for each lesson), FAQs (84% or more), and stories about PSP (84% or more). Many clients accessed additional resources; the most frequently accessed resources were on relationships with significant others (46/83, 55%), anger (42/83, 51%), communication skills (40/83, 48%), and problem solving and worry time (40/83, 48%). Most clients opted to receive therapist support once per week (74/83, 89%), but some opted to receive therapist support twice per week (6/83, 7%) or only on an as-needed basis (3/83, 4%). On average, clients sent 4.98 messages (SD 5.53) to their therapists and received 9.80 (SD 4.71) messages.

Some clients reported using the PSP Wellbeing Course concurrently with other treatments. At prescreen, some clients reported currently taking one or more medications for their mental health (18/83, 22%) or receiving mental health care from one or more providers (19/83, 23%), the most common of which were family doctors (13/83, 16%), psychologists (6/83, 7%), psychiatrists (4/83, 5%), and counsellors (3/83, 4%). Per the eligibility criteria described above, none of these clients were receiving mental health treatments from these providers more than twice per month. Several clients (4/83, 5%) indicated that they were on a waitlist to see a mental health care provider.

## Discussion

### Principal Findings

Canadian PSP experience high rates of mental health problems and face several barriers to mental health care [15,16]. ICBT is a promising solution for effectively managing symptoms of common mental disorders [19,20] in a private and convenient fashion that allows clients to overcome key barriers for care [15,17]. PSPNET has developed a tailored ICBT program for PSP called the PSP Wellbeing Course. This article presents the initial program outcomes.

The results indicated that the PSP Wellbeing Course is effective for treating symptoms of depression, anxiety, PTSD, panic disorder, and anger, and also improves clients’ functioning across three domains of life. The course appeared to be only slightly effective for treating symptoms of social anxiety, but this should be interpreted with caution because the subsample reporting clinically significant social anxiety was small (n=27). Nonetheless, in keeping with the learning health system approach [25], PSPNET will explore the possibility of providing additional resources to help clients manage social anxiety symptoms. This is important because approximately 15% of Canadian PSP—and 33% in this study’s treatment-seeking sample—struggle with clinically significant social anxiety [11]. Results concerning treatment satisfaction, working alliance, and engagement were promising. Although in our interviews with PSP stakeholders [15], most had expressed a preference for a flexible treatment duration, it was encouraging that most clients completed all lessons within 8 weeks. It was also very encouraging that working alliance was high because many PSP we interviewed previously [15] reported negative attitudes toward mental health care providers (eg, distrust, discomfort, beliefs that service providers do not understand PSP). Most clients accessed multiple additional resources, suggesting that they found them helpful and lending some support to recommendations for modularized, optional treatment elements in internet interventions [21].

Descriptively, these outcomes are comparable to the most recent published outcomes of the Wellbeing Course offered by the Online Therapy Unit to the general population of Saskatchewan [63]. Effect sizes, percentage changes, and reliable change rates were comparable for all available symptom measures with the possible exception of the SIAS-6/SPS-6, which appeared to show better outcomes for the Wellbeing Course. However, mean pretreatment social anxiety symptoms were considerably less severe in the present sample than in the Online Therapy Unit’s clients [63], and the smaller change in symptoms found in this study may be due, in part, to a floor effect. Of note, the present findings show that the PSP Wellbeing Course was moderately effective for treating anger, which has not been measured in research on the Wellbeing Course. PSP participating in this study reported concurrent use of psychotropic medication less frequently than clients of the Wellbeing Course [63]. Results concerning treatment satisfaction, working alliance, and engagement were similar across the two courses.

### Limitations

This study has several limitations. First, the data were collected during the COVID-19 pandemic, and the impact of the pandemic on outcomes is unclear. Second, there was no control condition and therefore no direct evidence that symptom change can be attributed to the PSP Wellbeing Course. However, it has been argued that the literature on digital mental health interventions would benefit from more research evaluating such interventions in real-world settings [64,65], as we did in this study. Third, at 8 weeks, we were missing PHQ-9 and GAD-7 data for 23% of clients and PCL-5 data for 25% of clients. A conservative approach was used to manage missing data, but it is possible that clients for whom data were missing may represent a unique subset of clients (eg, clients who experienced less positive outcomes).

### Future Directions

Further research can extend these findings in several ways. This article describes the results for only the first third of the sample (83 of 250) specified in our trial protocol; full results will be reported when available, including outcomes at 1-year follow-up and for PSPNET clients in Quebec. Further research will also be required to explore demographic and clinical predictors of treatment response for the PSP Wellbeing Course and to identify new ways to tailor and improve ICBT for PSP. Finally, although this study suggests that the PSP Wellbeing Course benefits the PSP who access it, economic evaluation will be required in the future.

### Conclusions

Preliminary results indicate that the PSP Wellbeing Course is a promising and effective method for treating symptoms of depression, anxiety, and PTSD among Saskatchewan PSP. Our results also suggest that the course improved clients’ functioning and reduced their symptoms of panic and anger, but not social anxiety. Clients reported good working alliance and treatment satisfaction, and they demonstrated good engagement. Consistent with the concept of learning health systems, PSPNET will continue applying research results to iteratively improve tailored ICBT services, such as by investigating ways to better treat symptoms of social anxiety among PSP. Our findings may also be helpful for other groups involved in researching, providing, funding, or otherwise supporting mental health care for this unique and underserved population.

## Abbreviations

DAR-5: Dimensions of Anger Reactions Scale
GAD-7: Generalized Anxiety Disorder-7
GEE: generalized estimating equations
ICBT: internet-delivered cognitive behavioral therapy
PCL-5: Posttraumatic Stress Disorder Checklist for the DSM-5
PDSS-SR: Panic Disorder Severity Scale Self-Report
PHQ-9: Patient Health Questionnaire-9
PSP: public safety personnel
PTSD: posttraumatic stress disorder
SDS: Sheehan Disability Scale
SIAS-6: 6-item Social Interaction Anxiety Scale
SPS-6: 6-item Social Phobia Scale
WAI-SR: Working Alliance Inventory-Short Revised
